# Non-Catalytic RISCs and Kinetics Determine Mammalian siRNA Sub-Cellular Localization

**DOI:** 10.1371/journal.pone.0143182

**Published:** 2015-12-23

**Authors:** Fengmin Ji, Lianyun Liu, Ya-Hsin Tien, Yi-Hsien Peng, Hoong-Chien Lee

**Affiliations:** 1 Institute of Biological Sciences and Biotechnology, Beijing Jiaotong University, Beijing 100044, China; 2 Department of Chemistry, Beijing Jiaotong University, Beijing 100044, China; 3 Department of Biomedical Sciences and Engineering, National Central University, Zhongli District, Taoyuan City 32001, Taiwan; 4 Department of Physics, Chung Yuan Christian University, Zhongli District, Taoyuan City 32023, Taiwan; 5 Center for Dynamical Biomarkers and Translational Medicine, National Central University, Zhongli District, Taoyuan City 32001, Taiwan; Wuhan University, CHINA

## Abstract

Small interfering RNAs (siRNAs) are fundamental to the regulation of cell function. Much is known about its gene interfering mechanism, but a kinetic description of it is still lacking. Here, we derived a set of reaction-diffusion equations for multiple RNA-induced silencing complex (RISC) pathways that give quantitative temporal and spatial descriptions of the siRNA process in mammalian cell, and are able to correctly describe all salient experimentally observed patterns of sub-cellular siRNA localization, including those that, at first glance, appear irreconcilable. These results suggest siRNA sub-cellular localization mainly concerns the non-catalytic RISC-target complex, and is caused by the selectiveness of RISC-target interaction and the permeability of the nuclear membrane to siRNA strands but not to RISC-target complexes. Our method is expected to be useful in devising RNAi based cell regulation strategies.

## Introduction

siRNA is a double-stranded RNA, 20–25 nucleotides in length that, through complementary base paring, sequence-specifically regulates cell functions including mRNA degradation and DNA methylation [1–3]. RNA interference (RNAi) was initially thought to be a form of post-transcriptional gene silencing with only a cytoplasmic pathway [4]. Later on, an siRNA nuclear pathway was discovered [5]. Experiments have shown that siRNA co-localizes with target RNA, but a molecular model capable of accounting for all observed patterns of siRNA localization [6–9] is lacking.

Argonaute (AGO) proteins—AGO1-4 in mammals—are key effectors in siRNA induced RNAi [10, 11]. In mammal siRNA action, an AGO protein loads the double stranded—the guide/antisense strand and the passenger/sense strand—siRNA, then discards one of the strands to forms a RNA-induced silencing complex (RISC). A guide-strand containing, or mature, RISC is guided by the strand to the target RNA and binds with it to form a complex, and either silences the target by AGO2-catalysed cleavage [10, 11], or otherwise disrupts its function by mRNA-destabilization [12, 14]. A passenger-strand RISC does not bind with the target and has no interference activity.

Ordinary differential equations (ODEs) have been used to describe the dynamics of the siRNA system [15–17]. Being temporal but not spatial in nature, these works did not address localization, which is a spatial phenomenon, nor did it address the fact that different RISCs have distinct pathways. Here, we developed a set of partial differential reaction-diffusion equations (PDEs) for the siRNA system based on the law of mass action and designed to yield realistic temporal and spatial simulations of multiple RISC pathways. We find that having multiple RISC pathways was necessary for understanding siRNA action, that siRNA sub-cellular localization is dominated by the localization of non-catalytic RISCs, and is a kinetic consequence of RISC-target interactions and the non-permeability of the nuclear membrane to RISC-target complexes.

In our simulation, the siRNA system is represented by two types of reaction equations, those describing the binding of the DNA strands for siRNA to the AGO proteins (*i* = 1 to 4) and the hybridization of the guide and passenger strands to form the double-stranded siRNA (*i* = 5):

$$\begin{array}{c} X_{i}+Y_{i}\overset{k_{i}}{\to }Z_{i}+U_{i};\;\left(i=1\;to\;5\right) \end{array}$$ (1)

where *X*_1−4_ = *D*, *X*_5_ = *G*, *Y*_1,2_ = *A*_*c*_, *Y*_3,4_ = *A*_*o*_, *Y*_5_ = *P*, *Z*_1_ = *R*_*cg*_, *Z*_2_ = *R*_*cp*_, *Z*_3_ = *R*_*og*_, *Z*_4_ = *R*_*op*_, *Z*_5_ = *D*, *U*_1,2,5_ = null, *U*_3_ = *P*, and *U*_4_ = G; and those describing the binding of RISCs to target RNA to form RISC-target complexes and, in the case of the complex *C*_*ct*_, its return to RISC after cleavage of target by the AGO2 protein:

$$\begin{array}{c} R_{cg}+T\overset{k_{6}}{\underset{k_{6}^{\prime }}{⇌}}C_{ct}\overset{k_{6}^{\prime \prime }}{\to }R_{cg};\;R_{og}+T\overset{k_{7}}{\underset{k_{7}^{\prime }}{⇌}}C_{ot} \end{array}$$ (2)

Abbreviations (lower case when used as subscripts) are: *D*, the double-stranded siRNA; *G*/*P*, guide/passenger strand of siRNA; *T*, target RNA (mRNA or nucleus RNA); *A*, AGO proteins; *R*, RISCs; *C*, RISC-target RNA complexes. The subscript *c* indicates the catalytic AGO2 protein; *o*, other AGO proteins.

From Eqs (1) and (2) we derive the set of 12 dynamic PDEs:

$$\begin{array}{c} \frac{dW_{i}}{dt}=F_{i}-\omega_{i}\nabla^{2}W_{i};\;\left(i=1\;\text{to}\;12\right) \end{array}$$ (3)

with the functions *F*_*i*_ and *W*_*i*_ defined in Table 1.

**Table 1:** Symbols and diffusion coefficients in Eq (3).

*i*	*W*_*i*_	*F*_*i*_ *	*ω*_*i*_(*μm* ^2^ *s* ^−1^)^*b*^
1	*D*	−*k*_*c*_ *A*_*c*_ *D* − *k*_*o*_ *A*_*o*_ *D* + *k*_5_ *GP*	30
2	*A*_*c*_	−*k*_*c*_ *A*_*c*_ *D*	20
3	*A*_*o*_	−*k*_*o*_ *A*_*o*_ *D*	20
4	*R*_*cg*_	*k*_1_ *A*_*c*_ *D* − *F*_10_	15
5	*R*_*cp*_	*k*_2_ *A*_*c*_ *D*	15
6	*R*_*og*_	*k*_3_ *A*_*o*_ *D* − *F*_11_	15
7	*R*_*op*_	*k*_4_ *A*_*o*_ *D*	15
8	*G*	*k*_4_ *A*_*o*_ *D* − *k*_5_ *GP*	40
9	*P*	*k*_3_ *A*_*o*_ *D* − *k*_5_ *GP*	40
10	*C*_*ct*_	$k_{6}R_{cg}T-\left(k_{6}^{\prime }+k_{6}^{\prime \prime }\right)C_{ct}$	0
11	*C*_*ot*_	$k_{7}R_{og}T-k_{7}^{\prime }C_{ot}$	0
12	*T*	$-\left(F_{10}+k_{6}^{\prime \prime }C_{ct}\right)-F_{11}-k_{8}T+S_{T}$	0

*k_1_ = *s*_*d*_ *k*_*c*_, *k*_2_ = (1-*s*_*d*_)*k*_*c*_, *k*_3_ = *s*_*d*_ *k*_*o*_, and *k*_4_ = (1-*s*_*d*_)*k*_*o*_, where *k*_*c*_ = *k*_*o*_ = 5 × 10^−3^ *nM* ^−1^ *s* ^−1^ and *s*_*d*_, ranging from 0 to 1, controls the relative abundance of guide and passenger strand; *k*_5_ = 2.8×10^−2^ *nM* ^−1^ *s* ^−1^, *k*_6_ = (*M*/*n*)$k_{6}^{\prime }$ = *k*_7_ = (*M*/*n*)$k_{7}^{\prime }$ = 10^−3^ *nM* ^−1^ *s* ^−1^, $k_{6}^{\prime \prime }$ = 7×10^−3^ *s* ^−1^, *k*_8_ = 4×10^−3^ *s* ^−1^.

The cell was modelled by a sphere of radius *r* = 10*μm* composed of a spherical nucleus of radius 0.45*r*, a cytoplasmic perinuclear shell of thickness 0.2*r*, and the outer shell. For simplicity the cytoplasm was composed of only the cytosol. The nucleus had a typical 1/10 volume of the cell and the perinuclear region had twice the volume of the nucleus.

The system was injected with fixed amounts of double-stranded siRNA and total AGO proteins initially: *D*(0) = 2*nM* in cytoplasm and *D*(0) = 0 in the nucleus (the notation *D*(0) denotes *D* at initial time with no spatial reference), and *A*_*c*_(0) + *A*_*o*_(0) = *A*(0) = 4*nM* throughout the entire cell, and was supplied with quantities of target RNA at a constant rate *S*_*T*_: *T*(0) = *T*_0_ and *S*_*T*_ = *T*_0_ *s* ^−1^, with *T*_0_ = 10*nM* and 2*nM*, either only in the nucleus (and zero elsewhere; case of nuclear target) or only in the perinuclear region (case of perinuclear target). All other quantities were initially set to zero, *W*_*i*_(0) = 0, *i* = 4 to 11, throughout the entire cell. The parameter *p*_*d*_∈[0, 1] controls the relative abundance of AGO2 versus non-AGO2 proteins: *A*_*c*_(0) = *p*_*d*_ *A*(0), *A*_*o*_(0) = (1-*p*_*d*_)*A*(0), and the parameter *s*_*d*_∈[0, 1] controls the relative abundance ofguide and passenger strands (Table 1). Values for the reaction coefficients (*k*’s) (Table 1) were derived from experiment [16] and those for diffusion coefficients were estimated based on the value of 27*μm* ^2^ *s* ^−1^ for the diffusion coefficient of GFP in eukaryotic cells [18] and letting the diffusion coefficient of a biology molecule be inversely proportional to the cube root of molecular weight. siRNA strands, AGO proteins and RISCs may diffuse across the (virtual) cytoplasm-nucleus boundary [9] (*ω*_*i*_≠0 in Table 1) while the target RNA (*T*) and target-containing complexes do not. In particular, this implies that non-AGO2 proteins are trapped in situ once they form *C*_*ot*_ complexes with target. The reaction rate *k*_8_ represents degradation of the target RNA by other unspecified enzymes.

## Results

In a typical round of computation, a set of values for *T*_0_, *s*_*d*_ and *p*_*d*_ were taken and the PDEs in Eq (3) were solved using the finite difference method for all sites in the cell from time zero to 120 minutes in one-second time steps separately for two cases: nuclear target and perinuclear target. All computation results are deposited at the public data storage site Figshare: http://dx.doi.org/10.6084/m9.figshare.1598072.

**Fig 1:**
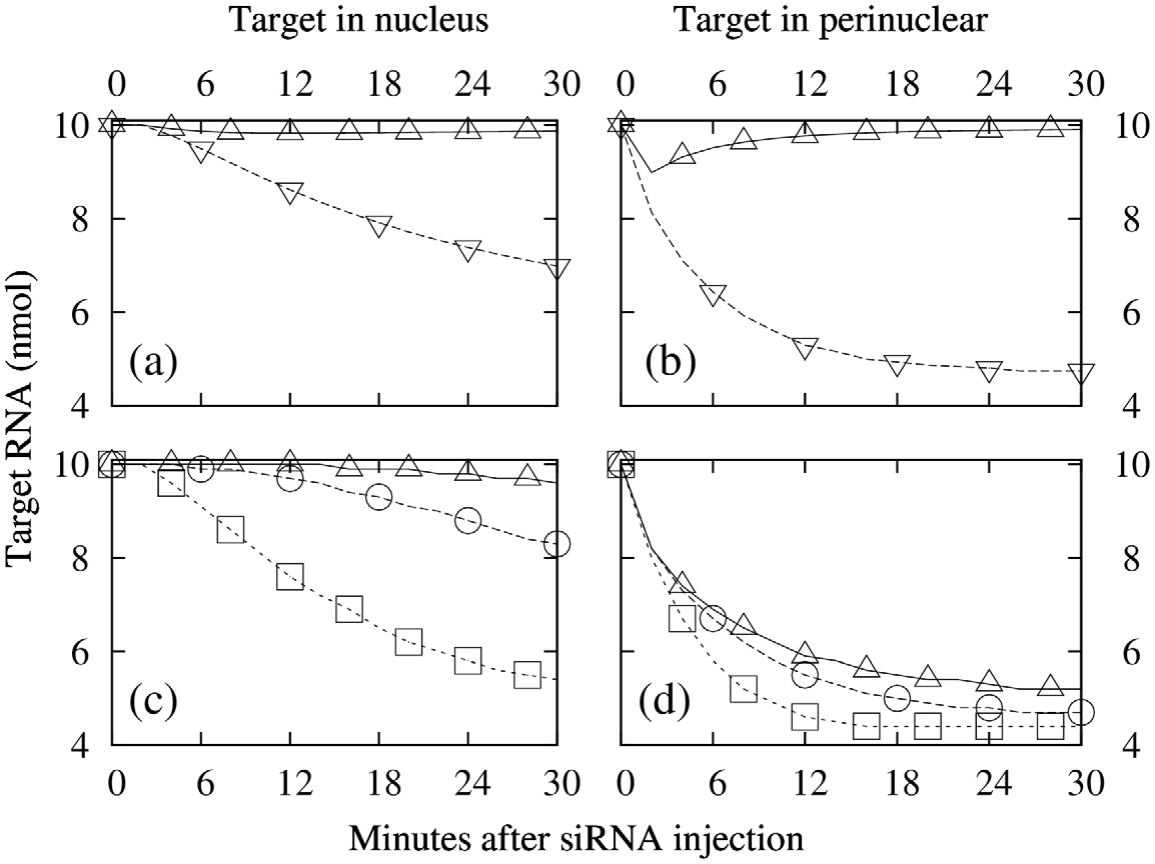
Time-evolution of target RNA concentration. (a) Average density, target in nucleus: △, *p*_*d*_ = 0, *s*_*d*_ = 1; ▽, *p*_*d*_ = 0.4, *s*_*d*_ = 0.6. (b) Same as (a), but with target in perinuclear region. (c) Target in nucleus at 0.29*R* (△), 0.35*R* (◯), and 0.42*R* (☐); *p*_*d*_ = 0.4 and *s*_*d*_ = 0.6; cell radius is *R* = 10*μ*M; nucleus-cytoplasm boundary is at 0.45*R*. (d) Same as (c), but target in perinuclear region at 0.48*R*, 0.55*R*, and 0.61*R*.

Time-evolution of the target RNA density after siRNA injection was sensitive to target location and the values of *s*_*d*_ and *p*_*d*_ (Fig 1). When there was no AGO2 protein (*p*_*d*_ = 0) to form RNA cleaving RISC, little loss of target RNA was observed regardless of target location, even when the production of the guide strand was maximized (*s*_*d*_ = 1), (△ in Fig 1(a) and 1(b)). Target depletion became noticeable when the amounts of AGO2 protein and guide strand were moderate (*p*_*d*_ = 0.4, *s*_*d*_ = 0.6). Rate of depletion was higher when target was in the perinuclear region than in the nucleus (▽ in Fig 1(a) and 1(b)). Location dependence was strong in the nucleus—the closer is the target to the nucleus-cytoplasm boundary the faster the depletion (Fig 1(c))—but weak in the perinuclear region (Fig 1(d)).

**Fig 2:**
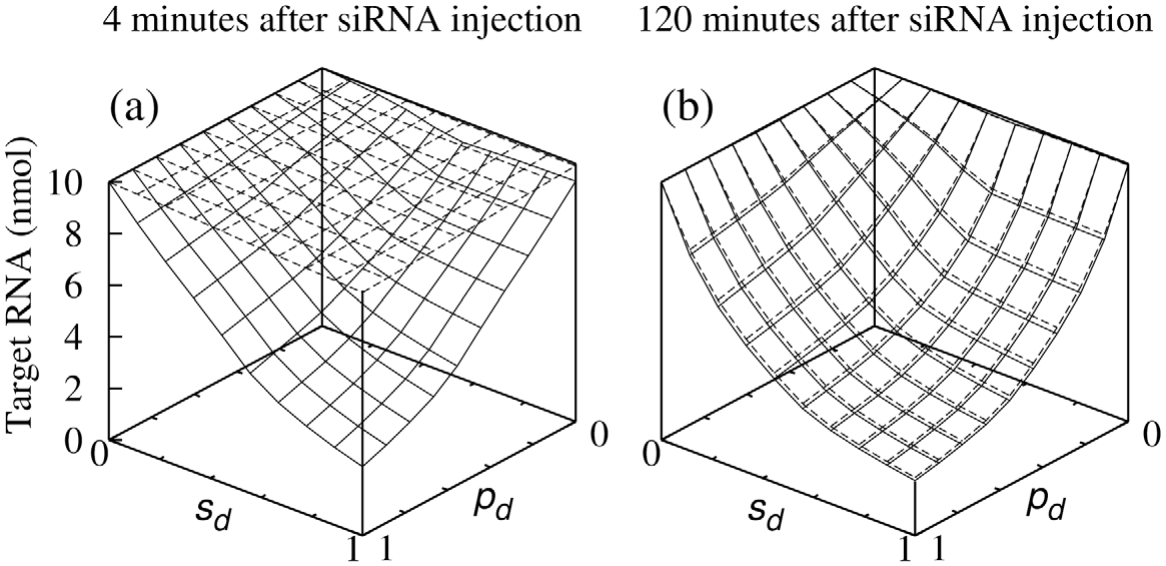
Density of target RNA as a function of *s*_*d*_ and *p*_*d*_. Density of target RNA (a) 4 minutes and (b) 2 hours (b) after siRNA injection. Dashed lines, target in nucleus; solid lines, target in perinuclear region. Values shown are averaged over region where target resides.

Response following siRNA injection was faster in the perinuclear region for all values of *p*_*d*_ and *s*_*d*_ (except when *p*_*d*_ = 0). At four minutes after siRNA injection, target depletion essentially reached equilibrium in perinuclear region but was minimal in the nucleus (Fig 2a). In both cases target depletion grew monotonically with increasing values of *p*_*d*_ and *s*_*d*_. Equilibrium was substantially achieved two hours after siRNA injection (Fig 2b).

**Fig 3:**
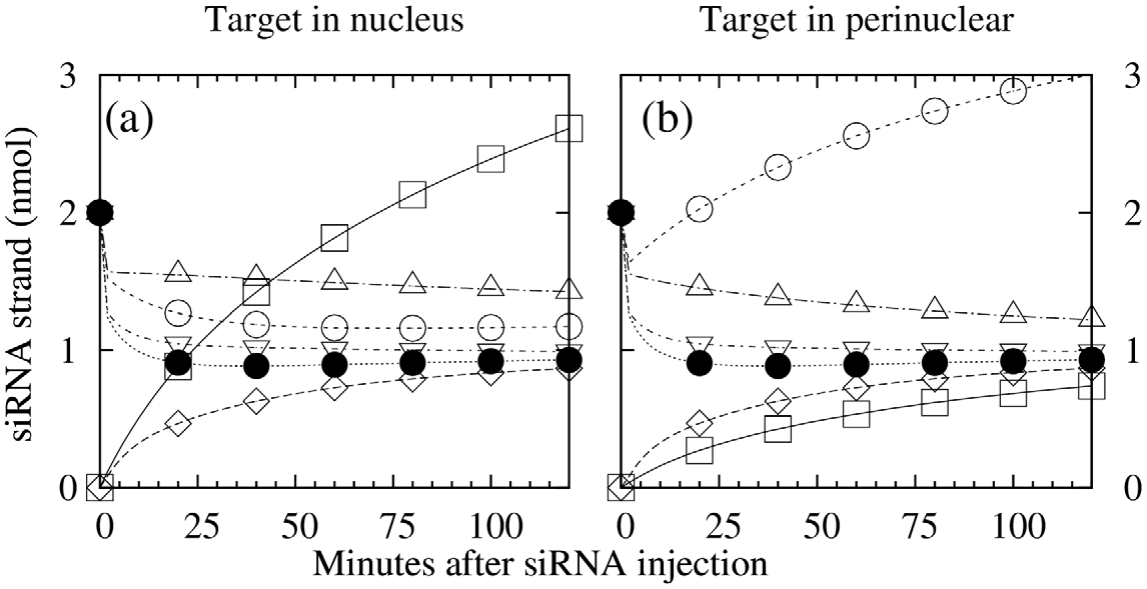
Time-evolution of guide and passenger strand densities, *D*_*g*_ and *D*_*p*_. (a) Target in the nucleus; (b) target in the perinucleus. Symbols ☐ (◊), ◯ (●), and △ (▽) stand for: guide (passenger) strand in nucleus, perinuclear region, and rest of cytoplasm, respectively. In all cases *p*_*d*_ = 0.4 and *s*_*d*_ = 0.6.

Time courses of the density of the passenger/sense strand *D*_*p*_ (= *D*+ *P*+ *R*_*cp*_+ *R*_*op*_) in all three regions, and that of the guide/antisense strand *D*_*g*_ (= *D*+ *G*+ *R*_*cg*_+ *R*_*og*_+ *C*_*ct*_+ *C*_*ot*_) in regions without target RNA, converged to a common value close to 1 nmol. In contrast, in the region where the target RNA resides, *D*_*g*_ increased with time to a value close to 3 nmol (Fig 3; ☐ in (a) and ◯ in (b)).

**Fig 4:**
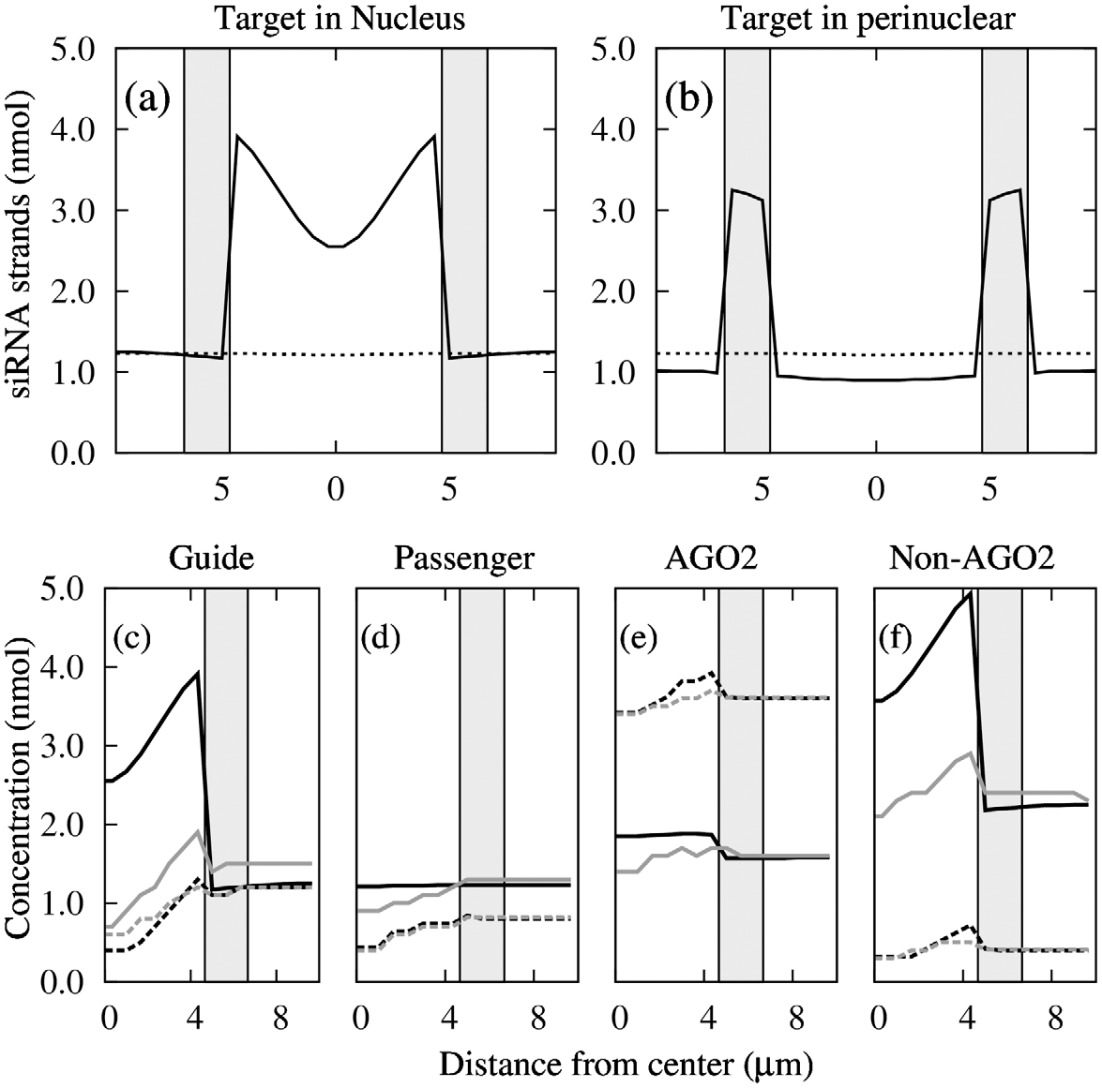
Density profiles of guide and passenger strands. Density profiles at *p*_*d*_ = 0.4 and *s*_*d*_ = 0.6, along the diameter in the case of (a) nuclear target and (b) cytoplasmic target; solid line, guide strand; dashed line, passenger strand. The two colored strips indicate the perinuclear region.

Density profile of the passenger strand (*D*_*p*_) along the cell diameter was low, about 1 nmol along the entire line (Fig 4), as was that of the guide strand (*D*_*g*_) except in the region where the target RNA was located. In the latter case, the density was about 3 to 4 nmol when it was in the nucleus (Fig 4(a)), and about 3 nmol when it was in the perinuclear region (Fig 4(b)).

**Fig 5:**
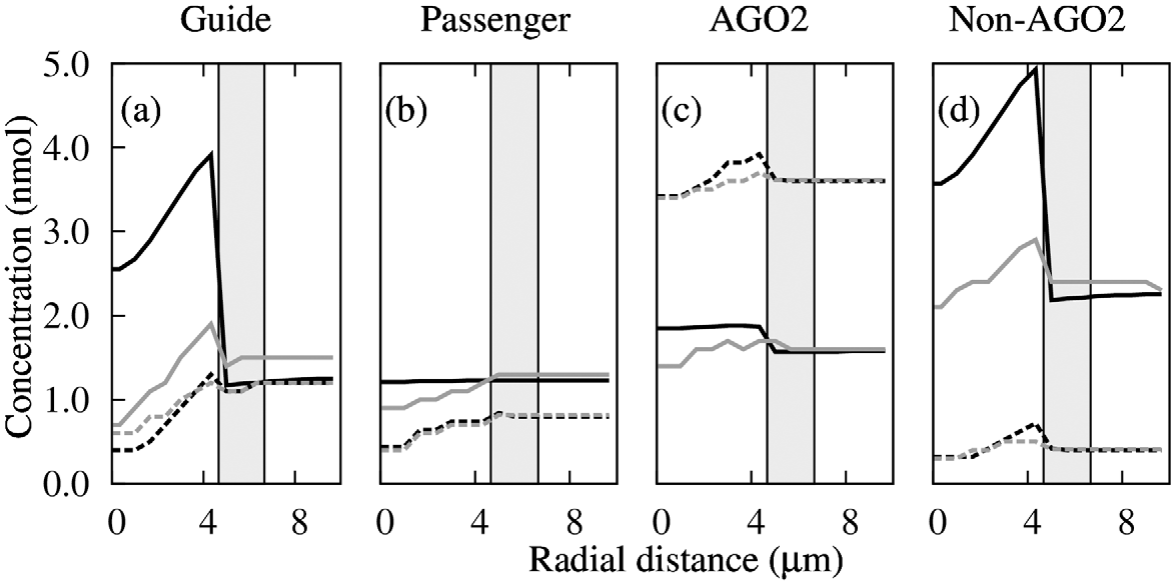
Radial density profiles. Radial density profiles of (a) guide strand, (b) passenger strand, (c) AGO2 protein, and (d) non-AGO proteins with target in the nucleus under four conditions: *T* = 10 nmol, *p*_*d*_ = 0.4 (solid black); *T* = 2 nmol, *p*_*d*_ = 0.4 (solid gray); *T* = 10 nmol, *p*_*d*_ = 0.9 (dashed black); *T* = 2 nmol, *p*_*d*_ = 0.9 (dashed gray). In all cases *s*_*d*_ = 0.6. Colored strip in panels indicate the perinuclear region.

Radial density profiles of the guide strand, the passenger strand, the AGO2 protein, and non-AGO proteins were distinct and responded differently to changing nuclear target conditions (Fig 5). Of the four cases, the passenger-strand profile is the most featureless (Fig 5(b)) because the strand (and RISCs bearing it) is blind to the presence of target.

## Discussion

A prominent difference between nuclear and perinuclear target is the speed of target attenuation after siRNA injection; fast for perinuclear target, and slow for nuclear target (Figs 1 and 2(a)). This is because siRNA is injected into, and RISCs are formed in, the cytoplasm, and the RISCs enter the nucleus only by diffusion [9], a slow process. Diffusion also explains why, in the case of nuclear target, response time is slow near the nucleus center while almost as fast as the case of perinuclear target near the nucleus-cytoplasm boundary (Fig 1(c)), and why in the perinulear region response time is largely location independent (Fig 1(d)).

The high concentration of guide strand in the region where the target RNA resides (Figs 3 and 4) is consistent with the siRNA localization reported in [6]. The enrichment of the guide strand is linked to target RNA because only guide strand-containing RISCs can form RISC-target complexes, and the non-catalytic complex *C*_*ot*_, once formed, stay in place because they neither breakup nor diffuse, and act as traps for guide strands. In contrast, the passenger strand does not bind to the target RNA and does accumulate around the target. The dip in the guide strand concentration near the nucleus center (Fig 4(a)) is likely caused by guide-strand bearing RISCs being consumed by target near the nucleus-cytoplasm boundary before they can diffuse to the center.

Imaging experiments have been conducted to examine the intracellular locations of two siRNA duplexes, 7SK siRNA targeting the small nuclear 7SK RNA and NS3 siRNA targeting the cytoplasmic HCV replicon mRNA [6]. In the case of cytoplasmic target our result (Fig 4(b)) essentially reproduces the experimental result (Fig 2f, [6]). In the case of nuclear target our result shows the localization of the guide strand in the nucleus (Fig 4(a)), whereas the experiment shows the localization of *both* guide and passenger (antisense and sense) strands, in the human Huh-7 and CHO cells (Figs 1f and 4f, [6]). These results led to the suggestion that cytoplasmic and nuclear RNAi pathways have distinct mechanisms, such that in the latter case both sense- and antisense-strand bearing RISCs are trapped in the nucleus [6].

The 7SK siRNA result observed in [6] can be explained without invoking distinct cytoplasmic and nuclear RNAi pathways: both the sense and antisense strands are guide strands, but they target RNAs from different sources. The antisense strand GGAGGUUUGUUCGAGAGUU targets the nuclear 7SK as supposed, while the sense strand AACUCUCGAACAAACCUCC can target the uncharacterised human ncRNA LOC101928306 (Sequence ID: ref|XR_251544.1|Range 32–50). The existence of two distinct nuclear targets for the 7SK siRNA can also explain why the two strands were observed to have different concentration profiles [6]. This hypothesis can be verified using human cells with either snRNA 7SK or ncRNA LOC101928306 is pre-silenced. Our search for a target for the sense strand in the NS3 siRNA yielded a null result.

Fluorescence correlation spectroscopic measurements of AGO2-RISC concentration in nuclear pathway of siRNA induced RNAi in ER239 cells revealed little AGO2-RISC accumulation in the nucleus [9]. Our results agree with these measurements: localization of AGO2 in the nucleus is weak and insensitive to target concentration and relative abundance (proportional to *p*_*d*_) of AGO2 protein (Fig 5(c)). Owing to the catalytic cleaving ability of the AGO2 protein, the target depleted AGO2-bearing complex does not trap the AGO2-RISC inside the nucleus, hence accumulation of AGO2 near the target is not expected.

For the case of nuclear target, our result attributing nuclear localization to the accumulate of non-catalytic (or non-AGO2, (Fig 5(d))), but not catalytic, RISC-target complexes reconciles the seemingly conflicting results reported in [6] (localization of guide strands, Fig 5(a))) and [9] (absence of localization of AGO2 protein, Fig 5(c))). It follows that the degree of localization is sensitive to target concentration (solid lines in Fig 5(d)) and relative abundance (proportional to (1-*p*_*d*_)) of non-AGO2 proteins (black lines in Fig 5(d)). These results can be experimentally validated.

It is now generally believed that in mammalian miRNA action, the predominant reason for reduced protein output in gene expression is the destabilzation of target RNA associated with miRNA induced mRNA deadenylation in the cytoplasm [13, 14]. This action, not caused by AGO endonuclease cleavage, is an issue separate from siRNA sub-cellular localization.

Our model gives a simple and unified molecular narrative for siRNA sub-cellular localization for both cytoplasmic and nuclear RNAi pathways that quantitatively reconciles seemingly conflicting experimental observations. Our work establishes localization as a phenomenon of non-catalytic RISC-target complex, a consequence of the selectiveness of RISC-target interaction and the non-diffusiveness of the RISC-target complex. The model provides a realistic framework for gaining deep insights in siRNA induced RNAi and for devising RNAi based cell regulation strategies.
